# The sexually dimorphic on the Y-chromosome gene (*sdY*) is a conserved male-specific Y-chromosome sequence in many salmonids

**DOI:** 10.1111/eva.12032

**Published:** 2012-12-03

**Authors:** Ayaka Yano, Barbara Nicol, Elodie Jouanno, Edwige Quillet, Alexis Fostier, René Guyomard, Yann Guiguen

**Affiliations:** 1 INRA, UR1037, LPGP, Fish Physiology and GenomicsRennes, France; 2 INRA, UMR1313, GABI, Domaine de VilvertJouy en Josas Cedex, France

**Keywords:** *sdY*, salmonids, sex-determining gene, sex-determination locus, molecular sexing

## Abstract

All salmonid species investigated to date have been characterized with a male heterogametic sex-determination system. However, as these species do not share any Y-chromosome conserved synteny, there remains a debate on whether they share a common master sex-determining gene. In this study, we investigated the extent of conservation and evolution of the rainbow trout (O*ncorhynchus mykiss*) master sex-determining gene, *sdY* (*sexually dimorphic on the Y-chromosome*), in 15 different species of salmonids. We found that the *sdY* sequence is highly conserved in all salmonids and that *sdY* is a male-specific Y-chromosome gene in the majority of these species. These findings demonstrate that most salmonids share a conserved sex-determining locus and also strongly suggest that *sdY* may be this conserved master sex-determining gene. However, in two whitefish species (subfamily Coregoninae), *sdY* was found both in males and females, suggesting that alternative sex-determination systems may have also evolved in this family. Based on the wide conservation of *sdY* as a male-specific Y-chromosome gene, efficient and easy molecular sexing techniques can now be developed that will be of great interest for studying these economically and environmentally important species.

## Introduction

Master sex-determining genes are the main genetic switches controlling the gonadal sex differentiation cascade leading to the development of ovaries or testes. In mammals, which harbor an XX/XY system, the *Sry* (s*ex-determining* r*egion of* the *Y chromosome*) gene was identified in the early 1990s (Sinclair et al. 1990) as the first vertebrate master sex-determining gene. In chickens, which have a female heterogametic (ZZ/ZW) sex-determination system, the Z-linked *Dmrt1* (*doublesex and mab-3 related transcription factor 1*) gene is a strong candidate for the chicken sex-determining gene (Smith et al. 2009), and this gene triggers gonad masculinization by a double dosage mechanism in males compared with females. Interestingly, *dmrt1* has also been shown to be conserved on the Z chromosome in the majority of bird species (Stiglec et al. 2007) including in the ancient ratite emu species (Shetty et al. 2002), suggesting a conservation of this gene as the master sex-determining gene in all bird species. In amphibians, the only currently known sex-determining gene is the *dm-W* gene in the African clawed frog, *Xenopus laevis* (Yoshimoto et al. 2008; Yoshimoto and Ito 2011). As this gene is located on the W female heterochromosome and evolved through the duplication of a *dmrt1* autosomal gene that lost its transactivation domain, this suggests that *dm-W* acts as a dominant negative regulator of *dmrt1* (Yoshimoto and Ito 2011). This evolution was, however, relatively recent because *dm-W* is conserved only in some *Xenopus* species and was most likely acquired after the divergence of the genera *Silurana* and *Xenopus* (Bewick et al. 2011). In teleost fish, an extremely high diversity in sex-determination systems and sex chromosomes has been found (Mank and Avise 2009), even within the same genus, as in the medaka (*Oryzias* spp.) (Takehana et al. 2007). Different master sex-determining genes have been recently characterized in teleosts including the *dmrt1* gene duplication, *dmrt1Y* in *Oryzias latipes and O. curvinotus* (Matsuda et al. 2002, 2007), the *gsdfY* (*gonadal soma*- *derived growth factor on the Y chromosome*) gene in *O. luzonensis* (Myosho et al. 2012), the *amhy* (*anti-Müllerian hormone on the Y*) gene in the Patagonian pejerrey, *Odontesthes hatcheri* (Hattori et al. 2012), the *amhr2* in the Tiger Pufferfish, *Takifugu rubrupes* (Kamiya et al. 2012), and the *sdY* (*sexually dimorphic on the Y-chromosome*) gene in the rainbow trout (Yano et al. 2012). To date, the evolution of these teleost master sex-determining genes has only been characterized in medaka species, in which the switch from one sex determinant to another has been found to be an evolutionarily recent event (Matsuda et al. 2003; Volff et al. 2003).

Salmonids sex-determination has been a long-standing mystery and also an important applied challenge, as many of these species are important both for aquaculture, in which sex control would be often beneficial, and for environmental purposes because of their emblematic value. Their sex-determination system is often described as always being male heterogametic (XX/XY) [reviewed in (Davidson et al. 2009)]. However, this assumption has been based only on the knowledge gained from the examination of a small subset of species, all belonging to the Salmoninae subfamily, i.e., in the genera *Oncorhynchus*, *Salmo,* and *Salvelinus* [reviewed in (Davidson et al. 2009)], whereas very little is currently known about sex-determination in the Coregoninae and nothing is known about sex-determination in the Thymallinae. In the majority of these species, the sex-determining (SEX) locus has been identified on different chromosomes (Woram et al. 2003; Li et al. 2011), leaving an open question about the uniqueness of a master sex-determining gene in this family (Davidson et al. 2009).

The recent discovery of *sdY* as the master sex-determining gene in rainbow trout (Yano et al. 2012) now allows this issue to be revisited. This is especially relevant because the *sdY* gene is included in the Y-chromosome genomic sequence of the rainbow trout (*OmyY1)*, which shares strong similarities with that of the Chinook salmon, *Oncorhynchus tshawytscha* (*OtY3*) (Brunelli et al. 2008), suggesting that *sdY* may be conserved as a Y-chromosome-specific sequence, at least within the *Oncorhynchus* genus. In this study, we thus investigated whether *sdY* is conserved as a male-specific Y-chromosome gene in other salmonids. Our results demonstrated that *sdY* is a male-specific conserved sequence found only in salmonid species and is tightly linked to the sex-determining (SEX) locus both in rainbow trout and brown trout, *Salmo trutta*, two salmoninae that are found in different genera. These results shed new light on salmonid sex-determination by demonstrating that the majority of salmonids do share a common sex-determination system (XX/XY) and a common SEX locus and that *sdY* may be this common master sex-determining gene.

Members of the salmonid family are present worldwide and many of them are species of major importance for aquaculture, wild stock fisheries, or recreational sport fisheries. Salmonids are also emblematic fish species of the Northern hemisphere and much concern has been raised about their preservation as many salmonid species are considered as threatened with extinction in many countries. This identification of a conserved master sex-determining gene in salmonids will then have important practical outcomes for molecular sexing. For instance, this will be of great interest for a better control of sex determination in aquaculture (and therefore improved artificial selection practices). Being able to unambiguously assess the sex of most salmonids (except coregonines) at any life stages will also contribute to facilitate and improve wild population surveys as well as stocking practices.

## Materials and methods

### Isolation of genomic DNA

Genomic DNA was extracted from fin clips of *Coregonus lavaretus*, *Coregonus clupeaformis*, *Stenodus leucichthys, Thymallus thymallus*, *Oncorhynchus mykiss*, *Oncorhynchus masou*, *Oncorhynchus tshawytscha*, *Salvelinus malma malma, Salvelinus alpinus*, *Salvelinus fontinalis*, *Salvelinus namaycush, Salmo trutta*, *Salmo salar*, *Hucho hucho*, *Parahucho perryi,* and *Esox lucius* preserved in 95% ethanol or dried on silica beads. All these animals were sexed based on macroscopic examination of the gonads or the emission of gametes during the active reproductive season. The number of samples per species, the locations of the collected samples, and the names of the collectors are listed in Table 1. Genomic DNA extractions were performed in 96-well plates using the Chelex® method as previously described (Gharbi et al. 2006). For each animal, a small portion of fin clip (2 mm × 2 mm) was placed in 100 μL of digestion solution, containing 5% of Chelex® 100 resin (Bio-Rad, Marnes-la-Coquette, France) and 0.5 μg/μL of proteinase K (Roche Diagnostics, Meylan, France) in distilled water. The samples were incubated at 55°C for 2 h, and then the proteinase K was inactivated at 95°C for 10 min. The plates were then briefly centrifuged to pellet the Chelex® resin, and the supernatant containing genomic DNA was stored at 4°C or −20°C.

**Table 1:** Origins of the samples and presence/absence of the *sdY* genotype in several salmonids

Subfamilies	Species	Common name	Location	Collector	*sdY* positive animals		Total

					Males	Females	
**Coregoninae**	*Coregonus lavaretus*	European whitefish	Lake Leman, Rhone, France	Alexis Champigneulle	8/8	9/9	17
	*Coregonus clupeaformis*	Lake whitefish	Lake Ontario, Ontario, Canada	Vance Trudeau	14/14	14/14	28
	*Stenodus leucichthys*	See fish	Innoko River, Alaska, United States	John Burr, Jeff Olsen, April Behr, Randy Brown	41/41	0/13	54
**Thymallinae**	*Thymallus thymallus*	Grayling	Pisciculture Saumon du Rhin, Obenheim, France	Martin Gerber	27/27	0/27	54
**Salmoninae**	*Oncorhynchus mykiss*	Rainbow trout	PEIMA, Sizun, France	A.Y and collaborators	218/218	0/207	425
	*Oncorhynchus masou*	Masu salmon	Field Science Center Oizumi Station, Yamanashi, Japan	Masaru Yagisawa, Goro Yoshizaki	5/5	0/5	10
	*Oncorhynchus tshawytscha*	Chinook Salmon	Big creek, Alaska, United States	Ora Schlei	27/27	1/41	68
	*Salvelinus malma malma*	Dolly Varden trout	Salmon River, Alaska, United States	Mark Lisac	20/20	0/20	40
	*Salvelinus alpinus*	Arctic char	Salmoniculture des Monts d'Arrée, Huelgoat, France	A.Y and collaborators	9/9	0/12	21
	*Salvelinus fontinalis*	Brook trout	Salmoniculture des Monts d'Arrée, Huelgoat, France	A.Y and collaborators	13/13	0/15	28
	*Salvelinus namaycush*	Lake char	Pisciculture Fédérale de Cauterets, Cauterets, France	Cauterets' Fish Hatchery Technicians	19/20	0/19	39
	*Salmo trutta*	Brown trout	PEIMA, Sizun, France	A.Y and collaborators	73/73	0/76	149
	*Salmo salar*	Atlantic salmon	River Loir, France	Guillaume Evanno	20/20	0/20	40
	*Hucho hucho*	Huchen	Polsh Anglers Association Hatchery, Lopuszna, Polland	Tomasz Mikolajczyk	10/10	0/10	20
	*Parahucho perryi*	Sakhalin taimen	Field Science Center Oizumi Station, Yamanashi, Japan	Masaru Yagisawa, Goro Yoshizaki	7/7	0/3	10
**Esocidae**	*Esox lucius*	Northern pike	Domaine de Lindre, Moselle, rue principale - 57260 Lindre-Basse, France	Thibaut Glasser, Julien Periz	0/10	0/10	20

### Cloning of the *sdY* sequences in various salmonid species

RNA extraction and reverse transcription reactions were performed as previously described (Govoroun et al. 2001) using testis samples collected from *O. masou*, *S. trutta*, *S. salar*, *P. perryi*, *T. thymallus*, *S. alpinus, S. fontinalis* and *C. lavaretus*. PCR reactions were performed using 0.5 μm of each primer, 1 μL of cDNA diluted 1:30, 200 μm dNTP mixture, and 2 μL 10× PCR Buffer (Sigma Aldrich Chimie s.a.r.l., Lyon, France) with 0.5 units of JumpStart *Taq* DNA Polymerase (Sigma Aldrich) in a total volume of 20 μL. Thermal cycling consisted of denaturation for 60 s at 94°C followed by 30 amplification cycles of 94°C for 30 s, 58°C for 30 s, and 72°C for 30 s, with a final extension of 3 min at 72°C. The primers used for RT-PCR are listed in Tables 2 and 3. These PCR products were directly used for sequencing. In *H. hucho*, the *sdY* gene was cloned and sequenced directly from genomic DNA using the Long PCR Product Sequencing (LoPPS) approach (Emonet et al. 2007). In *S. leucichthys*, the *sdY* gene was also PCR amplified from genomic DNA using several of the primer combinations that are listed in Tables 2 and 3. These PCR products were sequenced directly. Sequence alignments using partial sequences of *sdY* were processed using ClustalW. The sequence accession numbers for the proteins retained in the analyses are *C. auratus* Irf9, AFL69828; *S. salar* Irf9a, ACN11040; *O. mykiss* SdY, AB62689; *T. thymallus* SdY, JF826018; *S. trutta* SdY, JF826019; *S. salar* SdY, JF826020; *S. fontinalis* SdY, JF826021; *S. alpinus* SdY, JF826022; *O. masou* SdY, JF826023; *P. perryi* SdY*,* JF826024; *H. hucho* SdY*,* JF951962; *C. lavaretus* SdY, JF826025; and *S. leucichthys* SdY, JX196650. Rainbow trout Irf9a and b and Atlantic salmon Irf9b proteins were deduced from the following nucleotide sequences: CX258512, GE783444, EZ806353, CA346150, and CA375227.

**Table 2:** Names and combinations of the primers

**Primers used to characterize the *sdY* sequence in several salmonid species**		
Species	Primer Pair	
*Oncorhynchus masou*	sdY E1Sb	sdY E4AS1a
*Salmotrutta*	sdY E1Sb	sdY E4AS1a
*Salmosalar*	sdY E1Sb	sdY E4AS1a
*Parahuchoperryi*	sdY E1Sb	sdY E4AS1a
*Salvelinus alpinus*	sdY E1Sb	sdY E4AS1a
*Salvelinus fontinalis*	sdY E1Sb	sdY E4AS1a
*Coregonus lavaretus*	sdY E1Sb	sdY E4AS1a
*Huchohucho*	sdY E1Sa	sdY E4AS1a
*Parahuchoperryi*	sdY E1Sa	sdY E4AS1a
*Thymallusthymallus*	sdY E1Sb	sdY E4AS1a
*Stenodus leucichthys* (E1)	OmyY1Fw11022	OmyY1Rv11199
*Stenodus leucichthys* (E2)	OmyY1Fw11178	OmyY1Rv11918
*Stenodus leucichthys* (E3)	OmyY1Fw13771	OmyY1Rv13914
*Stenodus leucichthys* (E4)	OmyY1Fw15916	sdY E4AS1a
**Primers for genotyping *sdY* in several species**		
Species	Primer Pair		
*Coregonus lavaretus*	sdY E1S1	sdY E2AS4
*Coregonus clupeaformis*	sdY E1S1	sdY E2AS4
*Stenodus leucichthys*	sdY E2S1	sdY E2AS4
*Thymallus thymallus*	sdY E1S1	sdY E2AS4
*Oncorhynchus mykiss*	sdY E2S1	sdY E2AS2
*Oncorhynchus masou*	sdY E1S1	sdY E2AS5
*Oncorhynchus tshawytscha*	sdY E2S1	sdY E2AS4
*Salvelinus malma malma*	sdY E2S1	sdY E2AS4
*Salvelinusalpinus*	sdY E2S1	sdY E2AS4
*Salvelinus fontinalis*	sdY E2S1	sdY E2AS2
*Salvelinus namaycush*	sdY E2S1	sdY E2AS1
*Salmo trutta*	sdYE1S1	sdY E2AS4
*Salmo salar*	SS sdY S	SS sdY AS
*Hucho hucho*	HH sdY S	HH sdY AS
*Parahuchoperryi*	sdY E1S1	sdY E2AS4
all salmonid samples	18S S	18S AS
**Primers tested for amplification of *sdY* in Northern pike (*Esox lucius***)		
Species	Primer Pair	
*Esox lucius*	sdY E2S2	sdY E2AS2
*Esox lucius*	sdY E2S1	sdY E2AS1
*Esox lucius*	sdY E2S1	sdY E2AS3

**Table 3:** Primers sequence

Primers	Sequence
sdY E1S1	ATGGCTGACAGAGAGGCCAGAATCCAA
sdY E1Sa	CTGCCCTTCAATGGCTGACAGAGAG
sdY E1Sb	TTCAATGGCTGACAGAGAGGCCAGA
sdY E2S1	CCCAGCACTGTTTTCTTGTCTCA
sdY E2S2	GTGGAGTACTGCGAAGAGGAGGT
sdY E2AS1	TGCTCTCTGTTGAAGAGCATCAC
sdY E2AS2	CTGTTGAAGAGCATCACAGGGTC
sdY E2AS3	AGGAGACTGTGGCTTGGCTATG
sdY E2AS4	CTTAAAACCACTCCACCCTCCAT
sdY E2AS5	AGAGCATCACAGGGTCCACATCACG
sdY E4AS1a	GGGAGGACTCAAGCCAGATCCTGAA
HH sdY S	CCATGTCTGATCGTTTGAGGAAA
HH sdY AS	GCATAGATGCCTTCCTCCCTAGA
SS sdY S	GGCCTATGCATTTCTGATGTTGA
SS sdY AS	AGAGGATTGAACGGTCAGAGGAG
18S S	GTYCGAAGACGATCAGATACCGT
18S AS	CCGCATAACTAGTTAGCATGCCG
OmyY1Fw11022	TAKTTGAGTCCATCTGCCCTTCA
OmyY1Rv11199	TCAGACATGGAAATACCACAT
OmyY1Fw11178	TGTGGTTATTTCCATGCTGAT
OmyY1Rv11918	CACCRKTTTYTCAGGCATTAC
OmyY1Fw13771	TATTACTGACTCTGTGTGTGTCC
OmyY1Rv13914	TGAGTAAGAGAATCTGTACCG
OmyY1Fw15916	TGGTGTTGATTATAATTARATG

### PCR analysis for population and mapping family genotyping using the *sdY* sequence

Generally, PCR was performed using 0.1 μm of each primer, 40 ng of DNA, 200 μm dNTP mixture, and 2 μL of 10× PCR Buffer (Sigma Aldrich) with 0.5 units of JumpStart Taq DNA Polymerase (Sigma Aldrich) in a total volume of 20 μL. Thermal cycling consisted of denaturation for 60 sec at 94°C followed by 40 amplification cycles for *sdY* and 30 cycles for 18S:94°C for 30 s, 60°C for 30 s, and 72°C for 30 s, with a final extension of 3 min at 72°C. PCR products were electrophoresed on a 2% agarose gel. In addition, we tested the multiplex PCR analysis developed for rainbow trout (Yano et al. 2012) in *S. leucichthys*, *S. malma malma*, and *O. tshawytscha* as an easier molecular sexing tool allowing the simultaneous detection of both the *sdY* and *18S* genes. These multiplex PCR were performed using *sdY* primers, with *18S* primers as a positive amplification control. Each reaction contained 2 μL of a 1:5 dilution of Chelex®-extracted genomic DNA, 0.4 μm of each *sdY* primer (sdY E2S1 and sdY E2AS4; see Table 3 for the primer sequences), 0.1 μm of each *18S* primer (18S Fw and 18S Rv; see Table 3 for the primer sequences), 250 μm dNTP mixture, 1.5 mm MgCl_2_, 5× Green GoTaq Reaction Buffer (Promega France, Lyon, France), and 0.3 units of GoTaq polymerase (Promega), in a final volume of 12.5 μL. The following temperature cycling regime was applied: denaturation for 3 min at 95°C followed by 35 amplification cycles of 95°C for 30 s, 60°C for 30 s, and 72°C for 30 s, with a final extension of 3 min at 72°C. The PCR products were electrophoresed on 2% agarose gels.

### Characterization of *sdY* sex linkage groups in salmonids

In rainbow trout, two full-sib families of 46 individuals each were genotyped with *sdY* and markers previously assigned to RT01 (Guyomard et al. 2006). In brown trout, *sdY* mapping was performed on the same family panels as in (Gharbi et al. 2006). In these two species, centromeres were mapped using gynogenetic lines; DNA extraction, genotyping methods, marker accession numbers, map constructions, and graphic representation were all as previously described (Gharbi et al. 2006; Guyomard et al. 2006).

## Results

### *sdY* is a conserved sequence in salmonids

**Figure 1:**
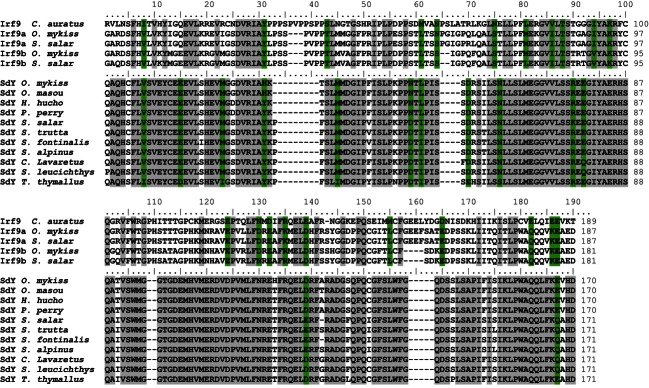
Alignment of Irf9 and SdY amino acid sequences in different salmonids. Using homology-based cloning, partial sequences of SdY were characterized in 11 representative species from the three salmonid subfamilies. ClustalW alignment of SdY deduced protein sequences reveals that SdY proteins are highly conserved in salmonids. Gray and green shading identify identical and similar amino acids, respectively. SdY sequences also show high homology with Irf9 sequences of different teleosts, and certain stretches of highly conserved amino-acids of the SdY proteins were also conserved in the Irf9 proteins. For SdY, identity and similarity are shown with respect to SdY sequences only and for Irf9 with respect to Sdy and Irf9 sequences. Sequence accession numbers for the proteins retained in the analyses are described in the Materials and methods section.

Using homology-based cloning with primers designed in the coding region of the rainbow trout *sdY* cDNA (Tables 2 and 3), we obtained partial *sdY* sequences in 10 additional salmonid species representative of the three salmonid subfamilies, i.e., Salmoninae (*O. masou*, *S. salar*, *S. trutta*, *H. hucho*, *P. perryi*, *S. alpinus*, and *S. fontinalis*), Thymallinae (*T. thymallus*), and Coregoninae (*C. lavaretus* and *S. leucichthys*). However, we were unable to obtain any *sdY-*related PCR products in the Northern pike (*E. lucius*)*,* which belongs to the Esocidae family and is considered to be the closest salmonid sister group (Lopez et al. 2004). ClustalW alignment of the deduced protein sequences revealed that SdY proteins are highly conserved (more than 87.1% identity) among salmonids (Fig. 1). As SdY shares some homology with the C-terminal domain of interferon regulatory factor 9 (Irf9) proteins (Yano et al. 2012), we also compared these SdY sequences with several fish Irf9 sequences. This comparison confirmed the relatively low identity of SdY with the C-terminal sequences of Irf9 (34.7–47.2%) but highlighted the fact that certain stretches of highly conserved amino-acids of the SdY proteins are also conserved in the Irf9 proteins (Fig. 1).

### The *sdY* gene is conserved as a male-specific Y-chromosome sequence in many salmonids

**Figure 2:**
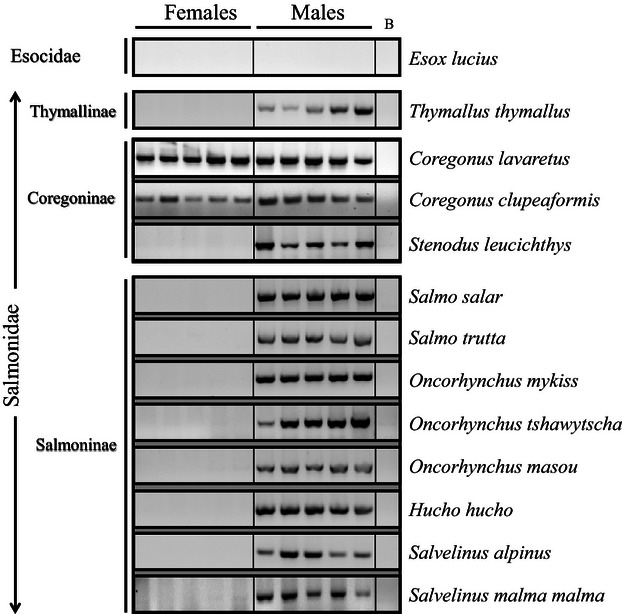
*sdY* sequence conservation in the male genomes of different salmonids.PCR amplification of *sdY* in male and female (*n* = 5) genomic DNA samples from different salmonid species showing a male-specific signal in Salmoninae and Thymallinae. In the Coregoninae species *C. lavaretus* and *C. clupeaformis*, *sdY* was detected both in male and female genomic DNA. No PCR products were obtained in Northern pike, *Esox Lucius* (Esocidae), which was chosen as an outgroup species closely related to salmonids.

**Figure 3:**
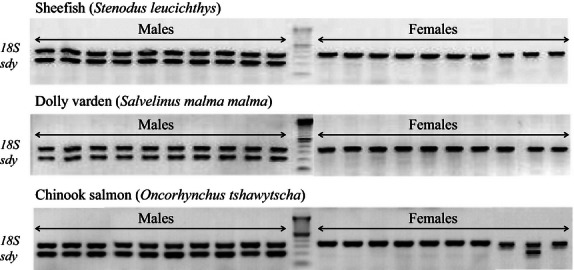
Multiplex PCR in *Stenodus leucichthys*, *Salvelinus malma malma*, and *Oncorhynchus tshawytscha*.PCR co-amplification of *sdY* and 18S (positive amplification control) in males and females genomic DNA samples (*n* = 10 animals/sex) from sheefish (*S. leucichthys*), dolly varden (*S. malma malma*) and Chinook salmon (*O. tshawytscha*). In the sheefish and dolly varden, *sdY* was strictly detected in males, whereas in Chinook salmon, *sdY* was detected in all males and also in a single female. The positive control 18S was detected in all PCR reactions.

*sdY* was strictly conserved as a male-specific sequence in nearly all Salmoninae members studied (*O. mykiss, O masou, S. salar*, *S. trutta*, *H. hucho*, *P. perryi*, *S. alpinus*, *S. fontinalis, and S. malma malma*) and in Thymallinae (*T. thymallus*); see Fig. 2. The only exceptions to this clear-cut distribution were one *sdY*-positive female out of 41 in *O. tshawytscha* (Fig. 3, Table) and one *sdY*-negative male out of 20 in *S. namaycush* (Table 1). However, in the Coregoninae subfamily, two different patterns emerged: in one species, *S. leucichthys*, *sdY* was clearly male specific; in two other species, *C. lavaretus* and *C. clupeaformis*, all males and all females were *sdY* positive (Fig. 2, Table 1). To facilitate the use of *sdY* as a potential molecular sexing tool, we tested a simple multiplex PCR assay that was initially developed for rainbow trout (Yano et al. 2012) to co-amplify *sdY* and a positive control, the *18S* gene. This multiplex PCR approach was tested in three different salmonid species (*S. leucichthys*, *S. malma malma*, and *O. tshawytscha*) and was found to be efficient and reliable (Fig. 3).

### *sdY* co-localizes with the phenotypic sex on the SEX linkage groups in both rainbow trout and brown trout

**Figure 4:**
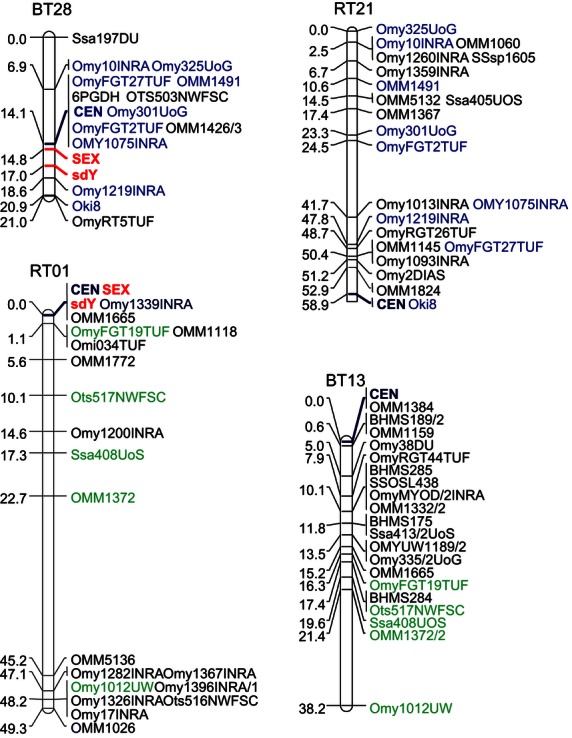
*sdY* and SEX-associated markers in rainbow trout (*Oncorhynchus mykiss*) and brown trout (*Salmo trutta*). Sex linkage groups (BT18, RT01) and their homologous counterparts resulting from the salmonid-specific whole-genome duplication (RT21 and BT13) are given in two Salmoninae species, i.e., brown trout (BT) and rainbow trout (RT). RT01 and BT18 share no conserved markers, with the notable exception of *sdY*, which is tightly linked with SEX in these two species. Common markers between BT28 and RT21 are shown in blue, and common markers between RT01 and BT13 are shown in green; CEN = centromere.

Genetic mapping of *sdY* along with several microsatellite SEX-linked markers in both rainbow trout (*O. mykiss*) and brown trout (*S. trutta*) demonstrates that *sdY* is tightly linked with the SEX locus on both rainbow trout linkage group 01 (RT01) and brown trout linkage group 18 (BT18); see Fig. 4. In brown trout, the 2.2 cM distance obtained between *sdY* and the phenotypic sex likely reflects a phenotyping error, most likely because the brown trout mapping families were less than 1-year old. This hypothesis was further confirmed when no recombination was found between *sdY* and the sexual phenotype in 150 individuals in a wild panmictic brown trout population. A comparison of the sex linkage groups BT18 in brown trout and RT01 in rainbow trout (Yano et al. 2012) demonstrated that RT01 and BT18 share no conserved markers, with the notable exception of *sdY*, and that the SEX locus surrounding markers from one species are all mapped on an autosomal group in the other species, BT28 with RT21 and RT01 with BT13 (Fig. 4).

## Discussion

It has been often suggested that genes involved in the sex differentiation pathway may have evolved independently as new master sex-determining genes in the course of vertebrate evolution (Schartl 2004; Marshall Graves and Peichel 2010). This idea has been mainly supported by the functional conservation of *dmrt1* as a master sex determinant in diverse vertebrate lineages (Yoshimoto et al. 2008; Herpin and Schartl 2011; Mawaribuchi et al. 2012). However, the recent finding that the rainbow trout master sex-determining gene, *sdY* (*sexually dimorphic on the Y-chromosome)* resulted from the *de novo* evolution of an immune-related gene demonstrated that there is likely an unexpected evolutionary plasticity in the mechanisms underlying vertebrate sex-determination (Yano et al. 2012).

**Figure 5:**
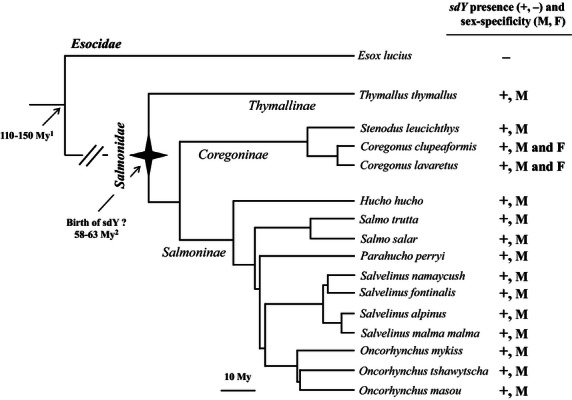
Phylogeny of salmonids and evolution of *sdY*. Phylogram of the species investigated in this study plotted with the presence (+)/absence (−) of *sdY* and its sex-specificity (M: male specific, M, F: present both in males and females. The salmonid phylogeny and the timing of divergence of species were based on Crête-lafrenière and collaborators (Crête-Lafreniere et al. 2012). Divergence time between Esocidae and Salmonidae was based on Near and collaborators (Near et al. 2012).

In this study, we investigated the extent of the conservation and evolution of *sdY* in the salmonid family. Based on a previously described similarity between two Salmoninae Y-chromosome-specific sequences, i.e., the rainbow trout *OmyY1* genomic sequence containing the *sdY* gene and the *OtY2* sequence found in Chinook salmon (Brunelli et al. 2008), we hypothesized that *sdY* may be conserved as a Y-chromosome gene, at least within the *Oncorhynchus* genus. Quite surprisingly, our results demonstrate that *sdY* is not only conserved as a male-specific Y-chromosome sequence in *Oncorhynchus* but also in many different salmonid species belonging to the *Salmoninae, Thymallinae,* and *Coregoninae* subfamilies (Fig. 5). We further confirmed this male-specific linkage in both rainbow trout and brown trout (*Salmo trutta*) by gene mapping experiments that demonstrated that *sdY* maps to the SEX linkage group in these two species. *sdY* is therefore the first and only sex marker known to be conserved among different salmonids. This conserved and tight male-specific linkage of *sdY* in salmonids contrasts with previous genetic mapping analyses showing that sex-linked markers in a given species always map to autosomal homologous linkage groups in other species (Woram et al. 2003; Li et al. 2011). This general lack of Y-chromosome synteny has often been used to suggest that salmonids most likely do not share the same master sex-determining gene (Davidson et al. 2009). Our results now provide clear evidence that the majority of salmonids do share the same master sex-determining locus and potentially also the same master sex-determining gene. These results also support one of the two hypotheses proposed by Woram and collaborators (Woram et al. 2003) suggesting that this gene has jumped into different ancestral autosomes during the evolution of salmonids, resulting into new Y sex chromosomes. Whether these chromosomal transitions were driven by transposition or translocation mechanisms is yet unknown, but the existence of several degenerate transposable elements found in the *OmyY1* sequence (Brunelli et al. 2008) a few kb upstream of *sdY* strongly suggests that the *sdY*/SEX locus has been transposed by moveable elements.

Our results provide an additional evidence that salmonid sex chromosomes are extremely ‘young’ (i.e., homomorphic) sex chromosomes in term of evolution and strongly support the jumping sex locus hypothesis proposed by Woram and collaborators (Woram et al. 2003). According to the ‘fountain of youth’ hypothesis (Perrin, 2009) young sex chromosomes would escape from degeneration by maintaining rare recombination events, that would be promoted by occasional spontaneous temperature-dependent sex inversions. The high-turnover model is an alternative hypothesis that postulates that the high turnover of master sex-determining genes in fish would enable the replacement of sex chromosomes before they begin to degenerate (Schartl 2004; Volff et al. 2007). In salmonids occasional spontaneous temperature-dependent sex inversion have been reported but most of them involved masculinization of genetic females (Craig et al. 1996; Azuma et al. 2004), thus not promoting sex inversion toward the female phenotype that has the highest rate of recombination. This does not fit well with the Perrin's ‘fountain of youth’ hypothesis. However, a new hypothesis can be proposed in the case of salmonids as a jumping sex locus like the sdY locus by continuously reshuffling the sex chromosomes would be also sufficient to prevent their decay. Interestingly this hypothesis shares with the high-turnover model the idea of a regular replacement of the sex chromosomes and keeps the ‘old-wine in-a-new-bottle’ Perrin's idea, that young sex chromosomes might actually harbor old sex-determining genes.

However, the male-specific sex linkage of *sdY* cannot be extended to all salmonids because we found at least two exceptions in the Coregoninae subfamily, with *sdY* being detected in both male and female genomes in *C. lavaretus* and *C. clupeaformis*. This absence of a sex-specific linkage of *sdY* in these Coregoninae may be the consequence of the existence of multiple sex chromosomes, as previously described by cytogenetic approaches in two other Coregoninae species, *C. albula* and *C. sardinella* (Frolov 1990; Jankun et al. 1991). In such cases, *sdY* may trigger testicular differentiation through a dosage mechanism, similarly to the *dmrt1* gene dosage hypothesis in chicken (Smith et al. 2009). However, other hypotheses may be proposed such like that females have a sex-specific inactive copy of *sdY*, similarly to the single sex-linked mutation reported in the *amhr2* gene in *Takifugu* (Kamiya et al. 2012) or that *sdY* is no longer the master sex determinant in these species. The latter hypothesis is supported by the fact that transition between different master sex-determining genes can arise within short evolutionary time periods, as observed in several medaka species (Matsuda et al. 2003; Volff et al. 2003).

Despite many attempts, we have been unable to clone any *sdY*-related sequence from Northern pike, *E. lucius*, which belongs to the Esocidae, a family that is considered to be the closest salmonid sister group (Lopez et al. 2004). This result is in agreement with a previous report (Yano et al. 2012) that showed that *sdY*-related sequences were absent from the teleost sequence databases (genome and expressed sequence tags databases), supporting the idea that *sdY* is a salmonid innovation. The birth of that master sex determining gene would then coincide with the age of the salmonid family (Fig. 5) that is dated around 60 million years old (Crête-Lafreniere et al. 2012).

Easy and reliable early identification of sex remains an important unsolved challenge in ecology and population genetics. From a practical point of view, molecular tools allowing accurate estimations of sex ratios would represent a considerable progress in the prediction of demographic evolution of stocks, which is an important concern in many salmonid species. From an academic point of view, the availability of such a technique would allow us to renew or greatly facilitate studies centered on or involving sex-ratio fluctuation, evolution or putative environmentally controlled sex reversion in natural populations. In population genetics, this ability offers the possibility of studying male-mediated versus female-mediated dispersion and introgression in natural populations following secondary contact (i.e., stocking or escapement from fish farms). Such a molecular sexing technique would also help to evaluate the impact of endocrine disruptors on complete or partial sex inversion in wild salmonid populations and enable a better management of sex control in salmonid aquaculture species, which is often an important issue for the production of all-female populations (Donaldson and Hunter 1982; Hunter and Donaldson 1983). As we demonstrated that *sdY* is both conserved in terms of sequence identity and also as a male-specific gene in many salmonids, this information can now be used to develop a simple and efficient molecular sexing technique that could be applied to many salmonid species. The main advantage of this sex-specific marker is its strong sequence conservation, which allows for the development of a unique test that can be used in multiple salmonid species. We provide here preliminary results indicating that this test could be realized quite easily, as we were able to accurately sex genotype different salmonid species from different subfamilies using a simple multiplex PCR approach that was initially designed for rainbow trout (Yano et al. 2012). However, caution should be taken before making any generalizations on applying these results to other species. In addition to *sdY* being present both in male and female genomes in several Coregoninae, we also found a single *sdY*-positive female in Chinook salmon (*O. tshawytscha*) and a single *sdY*-negative male in lake trout (*S. namaycush*). These exceptions to the rule may be due to human error in the assessment of the phenotypic sex of these animals, but these apparent discrepancies may also be biologically relevant; for example, the occurrence of ‘apparent’ XY females has been well-documented in Chinook salmon (Nagler et al. 2001; Williamson and May 2005; Williamson et al. 2008). In addition, the occurrence of potential XX males has also been reported in several salmonid species (Quillet et al. 2002; Metcalf and Gemmell 2006), and masculinization of genetic females by environmental factors or pollutants has also been demonstrated in other species (Craig et al. 1996; Azuma et al. 2004).

Taken together, our findings open new avenues for studying the molecular evolution of sex-determination and sex chromosomes in salmonids, including the simple assessment of male heterogamety in additional salmonid species, as shown in this study for the huchen (*H. hucho*), the Sakhalin taimen (*P. perryi*), the dolly Varden (*S. malma malma*), the European grayling (*T. thymallus*) and the Inconnu (*S. leucichthys*). These results also provide a biological basis for the development of new molecular sexing approaches that will be very important for ecology and ecotoxicology research and for better control of sex-determination in aquaculture in this economically and environmentally very important fish family.
